# TNFR2 deficiency impairs the growth of mouse colon cancer

**DOI:** 10.7150/ijbs.72606

**Published:** 2023-01-22

**Authors:** Ping Li, Yang Yang, Xinyu Yang, Yifei Wang, Chon-Kit Chou, Mengmeng Jiang, Jingbin Zheng, Fengyang Chen, Xin Chen

**Affiliations:** 1 State Key Laboratory of Quality Research in Chinese Medicine, Institute of Chinese Medical Science, University of Macau, Macau SAR, 999078, P.R. China.; 2 Department of Pharmaceutical Sciences, Faculty of Health Sciences, University of Macau, Macau SAR, China.; 3 MoE Frontiers Science Center for Precision Oncology, University of Macau, Macau SAR, China.

**Keywords:** Tumor necrosis factor (TNF), tumor necrosis factor receptor type II (TNFR2), colon cancer, cancer immunotherapy.

## Abstract

**Objective:** Tumor necrosis factor (TNF) receptor type II (TNFR2) is expressed by a wide spectrum of tumor cells including colon cancer, non-Hodgkin lymphoma, myeloma, renal carcinoma and ovarian cancer, and its exact role remains to be fully understood. In this study, we examined the effect of genetic ablation of TNFR2 on *in vitro* and *in vivo* growth of mouse MC38 and CT26 colon cancer cells.

**Methods:** CRISPR/Cas9 technology was used to knockout TNFR2 on mouse MC38 and CT26 colon cancer cells. *In vitro* growth and colony formation of wild-type (W.T.) and TNFR2 deficiency of MC38 and CT26 cells, as well as the potential mechanism, was studied. The growth of W.T. and TNFR2 deficient MC38 and CT26 tumors in mice and intratumoral CD8 CTLs were also examined.

**Results:** TNFR2 deficiency impaired *in vitro* proliferation and colony formation of cancer cells. This was associated with the inhibition of protein kinase B (AKT) phosphorylation and enhanced autophagy-induced cell death. Moreover, deficiency of TNFR2 also markedly impaired *in vivo* growth of MC38 or CT26 in the syngeneic C57BL/6 mice or BALB/c mice, respectively, accompanied by the decrease in soluble TNFR2 levels in the circulation and the increase in the number of tumor-infiltrating IFNγ^+^ CD8 cells.

**Conclusion:** TNFR2 plays a role in the growth of mouse colon cancers. Our study provides further experimental evidence to support the development of TNFR2 antagonistic agents in the treatment of cancer.

## Introduction

Aberrant high level of TNFR2 is expressed by a broad spectrum of human tumors, including colon cancer^1^, ovarian cancer^2^, breast cancer^3^,^4^, cholangiocarcinoma^5^,^6^, renal cell carcinoma^7^, colorectal cancer^8^,^9^, non-small cell lung cancer^10^, non-Hodgkin lymphoma^11^, and thoracic esophageal squamous cell carcinoma^12^. The clinicopathologic and prognostic implications of TNFR2 expression by tumor tissues were also reported. For example, TNFR2 expressed by breast cancer is associated with tumor size, clinical stage, and pathological grade^4^. TNFR2 expression by thoracic esophageal squamous cell carcinoma (ESCC) is positively related to invasion, clinical stage, and poor overall survival^12^. Furthermore, polymorphism of *TNFRSF1B*, the gene encoding TNFR2, is associated with the risk, survival, or response to the treatment of cancer patient^13^-^16^.

Consistent with the previous clinical investigations, studies based on human tumor cell lines also revealed the functional consequences of TNFR2 expression. For example, it was shown that TNFR2 promotes the growth of human SW480, COLO205, and HT29 colorectal cancer cells through PI3K-AKT signaling pathway or activation of STAT3^9^,^17^. Furthermore, in human MDA‑MB‑231 and MCF‑7 breast cancer cells, as well as Kapras299 and L428 lymphoma cells, TNFR2 expression promotes the growth of tumor cells and protects malignant cells from DNA damage caused by chemotherapeutic agent adriamycin^18^,^19^. TNFR2 also mediates the effect of TNF in promoting invasiveness of human CCKS1 cholangiocarcinoma cells^5^. In contrast, blockade of TNFR2 has been shown to induce the death of human OCCAR3 ovarian cancer cells^20^, and silencing of TNFR2 results in the inhibition of phosphorylation of AKT in HT29 cells^9^ and inhibits the proliferation of Karpas299 cells^19^. These *in vitro* studies advanced our understanding of the role of TNFR2 in the development, growth, metastasis and survival of human tumor cells. However, the role of TNFR2 in the *in vivo* growth of the tumor and the underlying mechanism remains elusive.

In order to further define the role of TNFR2 on tumor development, we used CRISPR/Cas9 technology to knock out TNFR2 gene in MC38 and CT26 colon cancer cells (designated as MC38/TNFR2^-/-^ and CT26/TNFR2^-/-^, respectively). The result showed that the growth of resultant MC38/TNFR2^-/-^ and CT26/TNFR2^-/-^ cells was markedly inhibited in both *in vitro* and *in vivo* settings. Furthermore, the deficiency of TNFR2 markedly reduced the phosphorylation of AKT and promoted autophagy in cancer cells. Interestingly, genetic ablation of TNFR2 markedly reduced the blood levels of soluble TNFR2 and increased the number of tumors infiltrating IFNγ^+^ CD8 CTLs. Furthermore, tumors in 15% MC38/TNFR2^-/-^ cell-bearing mice were completely regressed, and these mice had developed tumor-specific immunity. Therefore, our data support the notion that TNFR2 promotes tumor growth and thus represents a therapeutic target in the treatment of colon cancer.

## Materials and methods

### Mice, cells, and reagents

8-12 weeks old, female BALB/c mice and female C57BL/6 mice were initially purchased from Jackson Laboratory and maintained under specific pathogen-free in the Animal Facility of the University of Macau. The animal research protocol was given approval by the Animal Research Ethics Committee of the University of Macau.

The CT26 colon cancer cell line and Lewis lung carcinoma (LLC) were purchased from the American Type Culture Collection (ATCC). The MC38 colon cancer cell line was purchased from iCell Bioscience Inc. (Shanghai, China). Penicillin/streptomycin (P.S.), HEPES, fetal bovine serum (FBS), L-glutamine, RPMI 1640 medium, and high glucose DMEM medium were purchased from Gibco/ThermoScientific (Waltham, MA, USA). CT26 colon cancer cells were cultured in RPMI 1640 medium supplemented with 10% FBS, 1% P.S., 10 mM HEPES and 2 mM L-glutamine at 37 ^o^C and 5% carbon dioxide (CO2) in a humidified incubator. LLC and MC38 colon cancer cells were cultured in high glucose DMEM medium supplemented with 10% FBS and 1% P.S. at 37 ^o^C and 5% CO_2_ in a humidified incubator.

Antibodies of PerCP-Cy5.5 anti-mouse TCRβ (H57-597), APC rat anti-mouse CD8a (53-6.7), P.E. anti-mouse IFNγ (XMG1.2), PerCP-Cy5.5 anti-mouse CD4 (RM4-5), and P.E. anti-mouse CD120b (TNFR2, TR75-89) were purchased from BD Pharmingen (San Diego, CA). Antibodies of PE-Cy7 anti-mouse CD4 (GK1.5) and FITC anti-mouse CD45 (30-F11) were purchased from Invitrogen (eBioscience). Adenovirus Ad-mCherry-GFP-LC3 was purchased from Beyotime (#C3011, Shanghai, China). The polyclonal or monoclonal antibodies against Akt (#9272), Phospho-Akt (Ser473, #9271), β-actin (13E5, #4970), and LC3A/B antibody (#4108) were purchased from Cell Signaling Technology (Beverly, MA, USA); P62/SQSTM1 (18420-1-AP), Beclin 1 (11306-1-AP), GAPDH (10494-1-AP), and ATG5 (10181-2-AP) were purchased from Proteintech (Proteintech, Wuhan, China). LIVE/DEAD Fixable Near-IR Dead Cell Stain Kit and TRlzol® Reagents were purchased from Thermofisher Scientific. Mouse TNFR2 ELISA kits were purchased from Sino Biological Inc., China.

### Design of TNFR2 sgRNAs and generation of TNFR2-CRISPR/U^TM^ plasmids (based on CRISPR/Cas9 technology)

Guide RNA sequences for CRISPR/U^TM^ were designed by Guangzhou Ubigene Biosciences Co., Ltd (China). Two synthesized DNA oligos were inserted into pX330 CRISPR plasmid. After transformation and propagation, pX330 containing gRNA targeting TNFR2 (TNFR2-CRISPR/U^TM^ plasmid) were extracted with Plasmid Midi Kit (TransGen Biotech, Beijing, China) and verified with DNA sequencing.

### Generation of TNFR2 knockout cell line with CRISPR/Cas9 technology

TNFR2 knockout MC38 and CT26 cells were constructed by Guangzhou Ubigene Biosciences Co., Ltd (China). Briefly, the TNFR2-CRISPR/U^TM^ plasmid was sequenced to confirm that the sequence inserted was correct. At final, insert oligonucleotides for mouse TNFR2 gRNA1 (F): ACAAGCGTGCCACGCTGAAGAGG and TNFR2 gRNA2 (R): CTACTCAGTCCTCGCCAATGAGG were used in the following study. These two TNFR2 gRNAs were used to target the exon 2 of Tnfrsf1b gene (Figure S1). 3,000,000 MC38 and CT26 colon cancer cells were placed in a sterile tube and centrifuged at 300 g for 4 min, respectively. After the supernatant was discarded, the cells were resuspended with 600 μL Buffer R and mixed with 30 μg endotoxin-free TNFR2-CRISPR/U^TM^ plasmid. Add 3 ml Buffer E2 into the shock cup, and then put it into the card slot of the electrometer. The cells and plasmid mixture were extracted with 100 μL electrogun heads, inserted into the electroshock cup, and the electroshock was started under the electroshock condition. After the electrotransfer was completed, the cells were inoculated in a 6-well plate with preheated medium ready for further culture. 24-48 hours after transfection, the effect of electrotransfer was observed under the microscope, the wells with the best cell viability and percentage of green fluorescent cells were selected, and the medium containing puromycin was replaced for screening. After 2-3 days of screening, the cells were digested into a single-cell suspension for cell counting. A certain amount of cell suspension was diluted by the limited dilution method and inoculated into 96-well plates. The cells were incubated at 37 °C and 5% CO_2_ for static culture. Single-cell clones were screened out. The deletion of TNFR2 was confirmed by flow cytometry. The PCR products of the target site were ligated to T-Vector pMD™19 (Simple) (Takara) for DNA sequencing. Finally, two single-cell clones were expanded for inoculation in their respective* in vivo* tumor models.

### RNA Extraction and RT-PCR

Total RNA was extracted using the TRlzol®. Reagents from the cultivated cells according to the manufacturer's instructions. In this way, quantitative measurement was done using a Spectrophotometer NanoDrop^TM^ One Microvolume UV-Vis (ThermoFisher Scientific^TM^, USA). Additionally, cDNA was synthesized using PrimeScript™ RT Reagent Kit with gDNA Eraser (Perfect Real Time) (Takara Bio, USA) and C1000 Touch^TM^ Thermal Cycler (Bio-Rad, USA), and the total RNAs were considered as the template. PCR was performed using a ViiA7 Real-Time PCR System (ThermoFisher Scientific^TM^, USA) with β-actin as an internal control.

PCR conditions included a primary denaturation step at 95 °C for 5 min followed by 40 cycles of PCR (95 °C for 30 s, 58 °C for 30 s, 72 °C for 30 s) and 72 °C for 5 min. TNFR2 primer pairs were, forward: 5′-ACAGTGCCCGCCCAGGTTGTCTTG-3′ and reverse 5′-GCAGAAATGTTTCACATATTGGCCAGGAGG-3′. β-actin primer pairs were, forward: 5′-GCTTCTTTGCAGCTCCTTCGT-3′ and reverse 5′-CCTTCTGACCCATTCCCACC-3′.

### Cell proliferation assay

MC38/WT and MC38/TNFR2^-/-^ cells were counted and planted in 96-well plates with five replicates at 2,000 cells in 100 µL medium per well. CT26/WT and CT26/TNFR2^-/-^ cells were counted and planted in 96-well plates with five replicates at 1,500 cells in 100 µL medium per well. Cell viability was determined at the time indicated by using MTT assay. Optical density (O.D.) values were recorded at 570 nm by using the enzyme-labeled instrument. The cell growth curves were drawn according to O.D. values. The experiment was repeated three times.

### Colony formation assay

MC38/WT, MC38/TNFR2^-/-^ cells, CT26/WT, and CT26/TNFR2^-/-^ were counted and planted in 6-well plates at a density of 200 cells in 2 ml medium per well. The medium was refreshed every three days. This assay was terminated when two clones almost grew together. Cells were fixed using 4% paraformaldehyde for 20 min at room temperature and stained using crystal violet staining solution (Beyotime, Nantong, China). The quantities of clones were calculated by Image J. Colonies with > 50 cells were counted. The experiment was repeated three times.

### Western blotting

MC38/WT, MC38/TNFR2^-/-^, CT26/WT, and CT26/TNFR2^-/-^ cell lysates were extracted by immunoprecipitation (RIPA) lysis buffer (Beyotime, Shanghai, China) containing 1 μM PMSF and 1% cocktail at 4 ^o^C. The protein concentrations in each sample were determined using BCA protein assay kit (Thermo Scientific).

Each sample was denatured with 4 × protein loading buffer (Takara) at 100 °C for 10 min. Then, equal amounts of protein (20 μg per sample) were separated by 12% SDS-PAGE gel and transferred to polyvinylidene difluoride (PVDF, Bio-Rad, USA) membranes. After blocking for 1 h in 5% skim milk powder in PBST at room temperature, the membranes were incubated with the primary antibodies overnight at 4 °C and secondary antibodies for 1 h at room temperature. Protein bands were visualized by a hypersensitive chemiluminescence kit (Thermo Scientific) on ChemiDoc XPS system (Bio-Rad). Protein expressions were measured using the Bio-Rad Image Lab software 5.2.1.

### mCherry-GFP-LC3 assay

MC38/WT, MC38/TNFR2^-/-^ cells, CT26/WT, and CT26/TNFR2^-/-^ were seeded on the coverslips in 6-well plates. After 24 h, the cells were infected with 10 μL adenovirus Ad-mCherry-GFP-LC3 (multiplicity of infection (MOI) = 5) for 6 h according to the manufacturer's protocol. Cells were switched to fresh medium and incubated for an additional 24 h. Then, the cells were fixed and blocked for fluorescence detection. Analysis was performed using a laser confocal microscope (Leica SP8). For the cells infected with adenovirus Ad-mCherry-GFP-LC3, autophagosome and autolysosome status was evaluated by counting cells with GFP^+^/mCherry^+^-LC3 (yellow) and GFP^-^/mCherry^+^-LC3 (red) puncta, respectively. In the tandem mCherry-GFP-LC3 assay, when an autophagosome fuses with the lysosome to form an autolysosome under acidic environments, GFP fluorescence is quenched in the autolysosome, whereas mCherry fluorescence is more stable. Therefore, when an autophagosome has not yet fused with a lysosome or when the degradation's function of the lysosome with acidification is impaired, co-localization of both GFP and mCherry fluorescence shows GFP^+^/mCherry^+^-LC3 (yellow) puncta in the merged image. In contrast, mCherry alone (without GFP) fluorescence presents GFP^-^/mCherry^+^-LC3 (red) puncta, which corresponds to an autolysosome.

### Tumor inoculation and separation of tumor-infiltrating leukocytes

MC38/WT and MC38/TNFR2^-/-^ were subcutaneously injected into the right flank of C57BL/6 mice in single-cell suspension with 500,000 cells in 0.1 ml of PBS per mouse. CT26/WT and CT26/TNFR2^-/-^ tumor cells were subcutaneously injected into the right flank of BALB/c mice in single-cell suspension with 200,000 cells in 0.1 ml of PBS per mouse. After indicated times, all the lymphoid tissue was pushed through a 70-μm pore size cell strainer to create a single-cell suspension. In some experiments, tumor-free mice were reinoculated with MC38/WT cells into the right flank, and the same number of LLC tumor cells were subcutaneously injected into the left flank. Tumor size was calculated by the following formula: (length×width^2^)/2. "Survival" represents the time to develop a 4 cm^3^ tumor or a moribund state, a humane endpoint that triggers euthanasia.

### ELISA for soluble TNFR2

Soluble TNFR2 levels in serum were determined using ELISA kit (Sino Biologicals Inc. PA, SEK50128). Samples were processed according to the manufacturer's instructions.

### Flow Cytometry

After blocking FcR, cells were incubated with appropriately diluted antibodies and finally suspended in FACS buffer for cytometric analysis. The acquisition was performed by BD LSRFortessa and BD FACSAria™ Fusion flow cytometer. For intracellular cytokine staining, cells were restimulated with ionomycin, PMA, and BD GlogiPlug for 5 h. Data analysis was conducted by using FlowJo software (Tree Star Inc., Ashland, OR). FACS analysis was gated on the live cells only by using a LIVE/DEAD Fixable Dead Cell Stain Kit (Life Technologies).

### Statistical Analysis

All data were presented as means ± SEM, and statistically significant differences between groups were performed by a two-tailed, paired Student *t* test, or ANOVA. All statistical analysis was performed with GraphPad Prism 7.0. A P value < 0.05 was considered to be statistically significant.

## Results

### Generation of TNFR2 knockout MC38 and CT26 colon cancer cell lines

**Figure 1:**
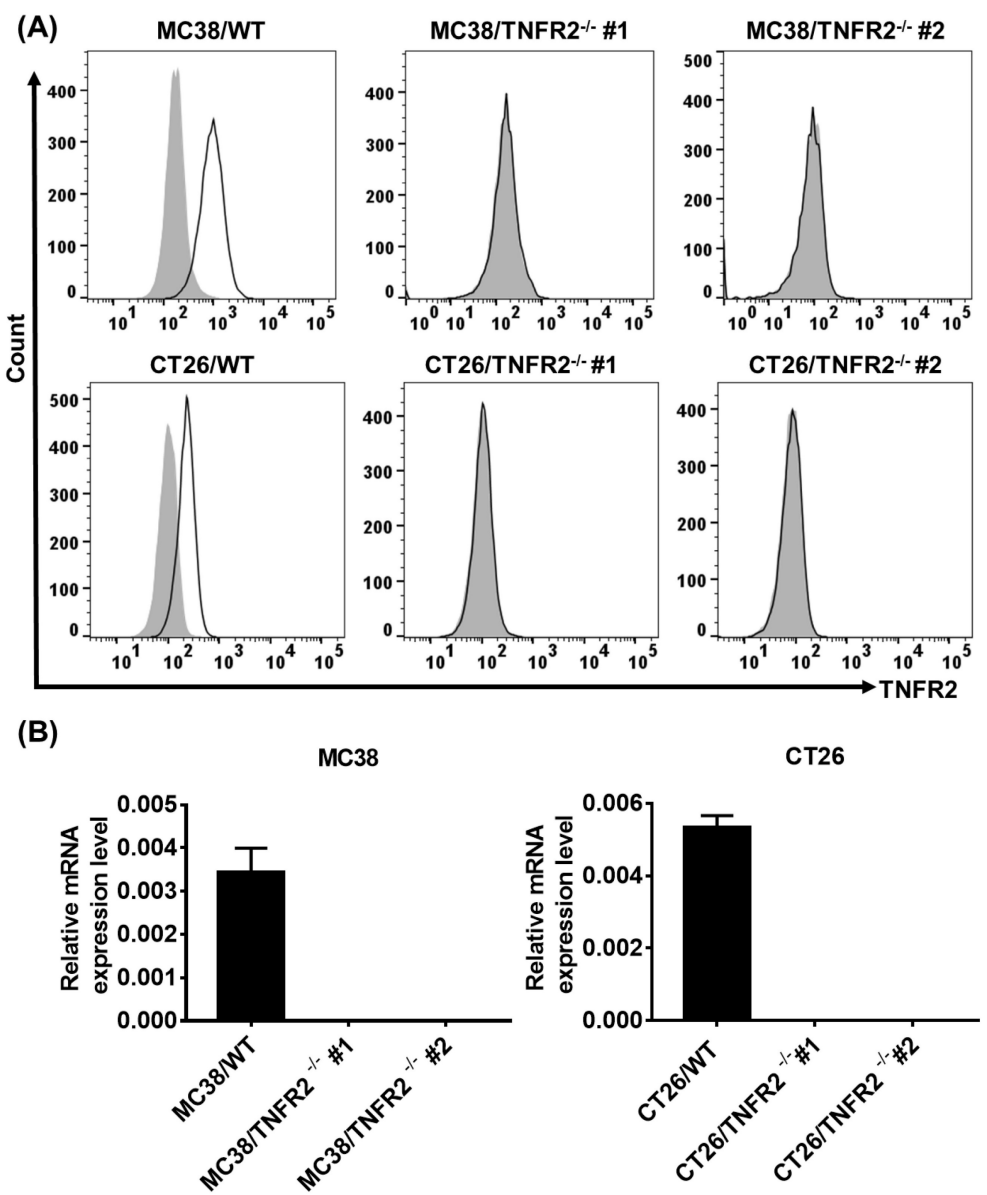
** Generation and identification of TNFR2-knockout MC38 and CT26 colon cancer cells using the CRISPR/Cas9 system. (A)** Flow cytometry analysis of TNFR2 expression on the TNFR2 knockout (TNFR2^-/-^) MC38 and CT26 cell lines vs. corresponding control W.T. cell lines. The solid line represents anti-TNFR2 antibody staining; the gray-shaded histogram represents the isotype control. **(B)** Real-time PCR analysis of TNFR2 derived from control and TNFR2^-/-^ cell lines. The data shown are representatives of at least three separate experiments with similar results.

To generate CRISPR/Cas9 plasmid containing gRNAs specific targeting the second exon of the Tnfrsf1b gene (NCBI Gene ID: 21938), two sgRNAs were inserted into pX330-based plasmid and confirmed with DNA sequencing (data not shown). MC38/WT and CT26/WT cells were transiently transfected with TNFR2-pX330 plasmid by electrotransfer, and the cells were screened by puromycin. After selecting single-cell clones, we analyzed the nucleotide sequences of the PCR products of target DNA and confirmed the indel mutations were introduced into the genome (Supplementary Figure S2). Deficiency in TNFR2 expression of these two clones was also confirmed by FACS (Figure 1A) and real-time PCR (Figure 1B). These clones were used in subsequent studies.

### *In vitro* proliferation and clone formation of TNFR2-deficient cells are impaired

**Figure 2:**
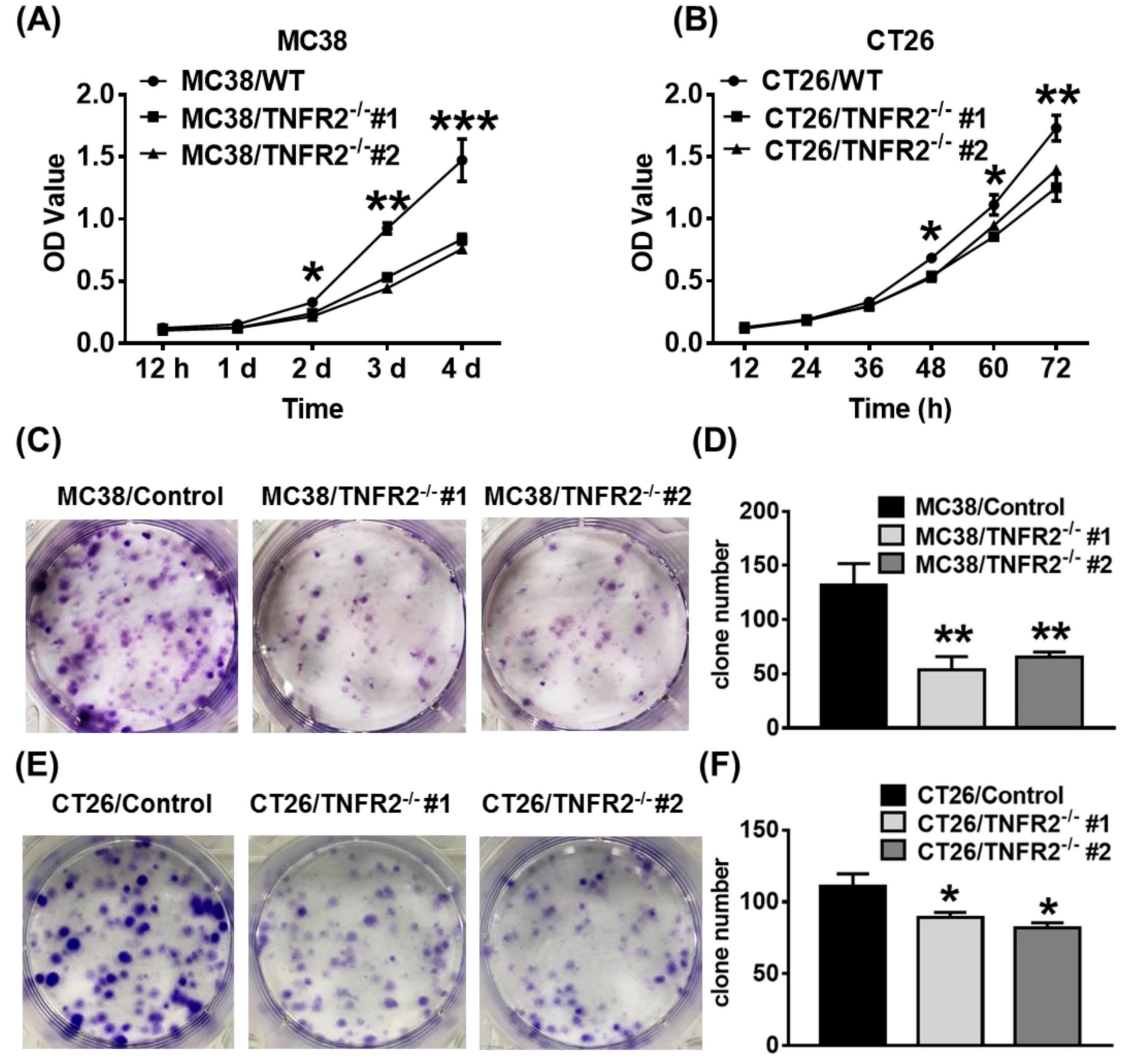
** TNFR2 deficiency impairs *in vitro* growth of MC38 and CT26 colon cancer cells.** MC38/WT and MC38/TNFR2^-/-^ cells were counted and planted in 96-well plates with five replicates at 2,000 cells in 100 µL medium per well. CT26/WT and CT26/TNFR2^-/-^ cells were counted and planted in 96-well plates with five replicates at 1,500 cells in 100 µL medium per well. The proliferation of cells was determined by MTT assay at the time indicated. CT26/WT, CT26/TNFR2^-/-^, MC38/WT, and MC38/TNFR2^-/-^ cells were counted and planted in 6-well plates at a density of 200 cells in 2 ml medium per well. The medium was refreshed every 3 days. This assay was terminated when two clones almost grew together, and the number of clone formations in the culture was counted. Growth curves **(A-B)**, image **(C and E)**, number of clone formation **(D and F)** of MC38/WT, MC38/TNFR2^-/-^, CT26/WT, and CT26/TNFR2^-/-^ cells. Data (means ± SEM, *N* = 3) shown are representatives of three separate experiments with similar results. By comparison with indicated groups, *p < 0.05, **P < 0.01, ***P < 0.001.

We next examined the effect of deficiency of TNFR2 on *in vitro* growth and clone formation of tumor cells. As shown in Figure 2A-B, TNFR2 ablation markedly decreased the proliferation of MC38 and CT26 cells (*P* < 0.05-0.001), and on day 4 or day 3 of culture, the proliferation was inhibited by 43% and 24%, respectively. Furthermore, colony formation of MC38 and CT26 cells was also reduced by 50% and 23% (Figure 2C-F, *P* < 0.05-0.01). Therefore, deficiency of TNFR2 impairs the growth and clone formation of MC38 and CT26 cells.

### Deficiency of TNFR2 results in the reduced phosphorylation of AKT

**Figure 3:**
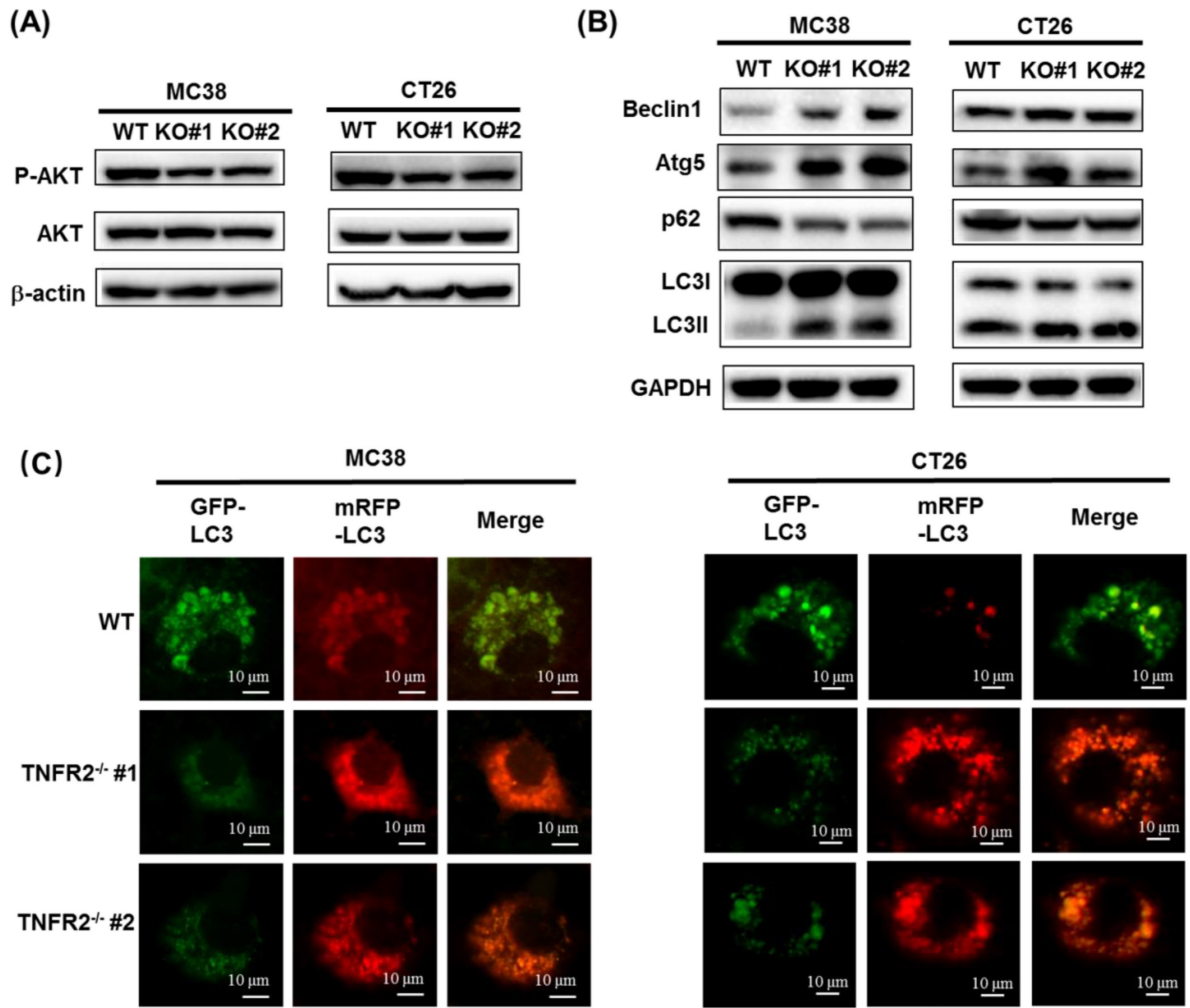
** The deficiency of TNFR2 reduces phosphorylation of AKT and promotes autophagy. (A)** Western blot analysis of P-AKT, AKT, and β-actin. The lysates derived from MC38/WT, MC38/TNFR2^-/-^#1 (KO #1), MC38/TNFR2^-/-^#1 (KO #2); CT26/WT, CT26/TNFR2^-/-^#1 (KO #1), and CT26/TNFR2^-/-^#2 (KO #2) cells were immunoblotted with a panel of antibodies specific for P-AKT, AKT, and β-actin (a loading control), respectively. The representative figures of the western blot assay were shown. **(B)** Western blot analysis of LC3, Beclin 1, p62, Atg5, and GAPDH. The lysates derived from MC38/WT, MC38/TNFR2^-/-^#1 (KO #1), MC38/TNFR2^-/-^#1 (KO #2); CT26/WT, CT26/TNFR2^-/-^#1 (KO #1), and CT26/TNFR2^-/-^#2 (KO #2) cells were immunoblotted with a panel of antibodies specific for LC3, Beclin 1, p62, Atg5, and GAPDH (a loading control), respectively. The representative figures of the Western blot assay were shown. **(C)** MC38/WT, MC38/TNFR2^-/-^#1, and MC38/TNFR2^-/-^#2; CT26/WT, CT26/TNFR2^-/-^#1, and CT26/TNFR2^-/-^#2 cells were infected with Ad-mCherry-GFP-LC3 at the multiplicity of infection (MOI) = 5, respectively. The mRFP-LC3 and GFP-LC3 puncta were examined by using a confocal microscope. Scale bar =10 μm. The data shown are representatives of at least three separate experiments with similar results.

The PI3K/AKT is one of the critical dysregulated signaling pathways in different cancer cells, including colorectal cancer (CRC), and its abnormal activation can result in increased proliferation, invasion, and migration of cancer cells^21^. We found that the deficiency of TNFR2 in MC38 and CT26 cells caused the inhibition of the phosphorylation of AKT (Figure 3A, Figure S6), whereas the phosphorylation of ERK, JNK, and P38 and total protein levels of them did not change (data not shown). Our data are consistent with the result of the previous study based on the silence of TNFR2 in HT29 cells^9^. Therefore, PI3K/AKT may be an important component of TNFR2 signaling pathway in promoting the proliferation of colon cancer cells.

### The deficiency of TNFR2 activates autophagy

A previous study reported that the induction of autophagy and apoptosis could inhibit the growth of CRC cells^22^. It was also reported that PI3K/AKT/mTOR signaling pathway plays a critical role in regulating autophagy and apoptosis in CRC^23^,^24^. Although we could confirm that TNFR2 deficiency resulted in a marked decrease in the phosphorylation of AKT, we failed to observe that deficiency of TNFR2 increased the apoptosis of cancer cells (data not shown). To examine the effect of TNFR2 deficiency on autophagy of mouse colon cancer, we analyzed the autophagy-related hallmarks including Beclin1, LC3, Atg5, and p62/SQSTM1 by Western Blotting. Results showed that the deficiency of TNFR2 on MC38 and CT26 colon cancer cells markedly decreased p62/SQSTM1 expression while increased the expression of Beclin1, Atg5, and LC3-II/LC3-I (Figure 3B, Figure S6). Further, the subcellular location of LC3, mainly in the autophagosomes, was detected by using a confocal microscope after infecting a report vector Ad-mCherry-GFP-LC3 in both W.T. and TNFR2 knockout MC38 or CT26 cells. The results demonstrated that MC38/TNFR2^-/-^ and CT26/TNFR2^-/-^ cells expressed GFP^-^/mCherry^+^-LC3 (red) puncta (Figure 3C), indicating that autophagosome and lysosome were fused. The autolysosomes present red puncta because GFP fluorescence is more rapidly quenched by low lysosomal pH, whereas mCherry fluorescence is more stable. Thus, in addition to decreased AKT phosphorylation, the increased autophagy of TNFR2-deficient MC38 and CT26 colon cancer cells may also be attributable to the inhibition of growth of these cells.

### Deficiency in TNFR2 impairs the growth of tumors in normal mice

**Figure 4:**
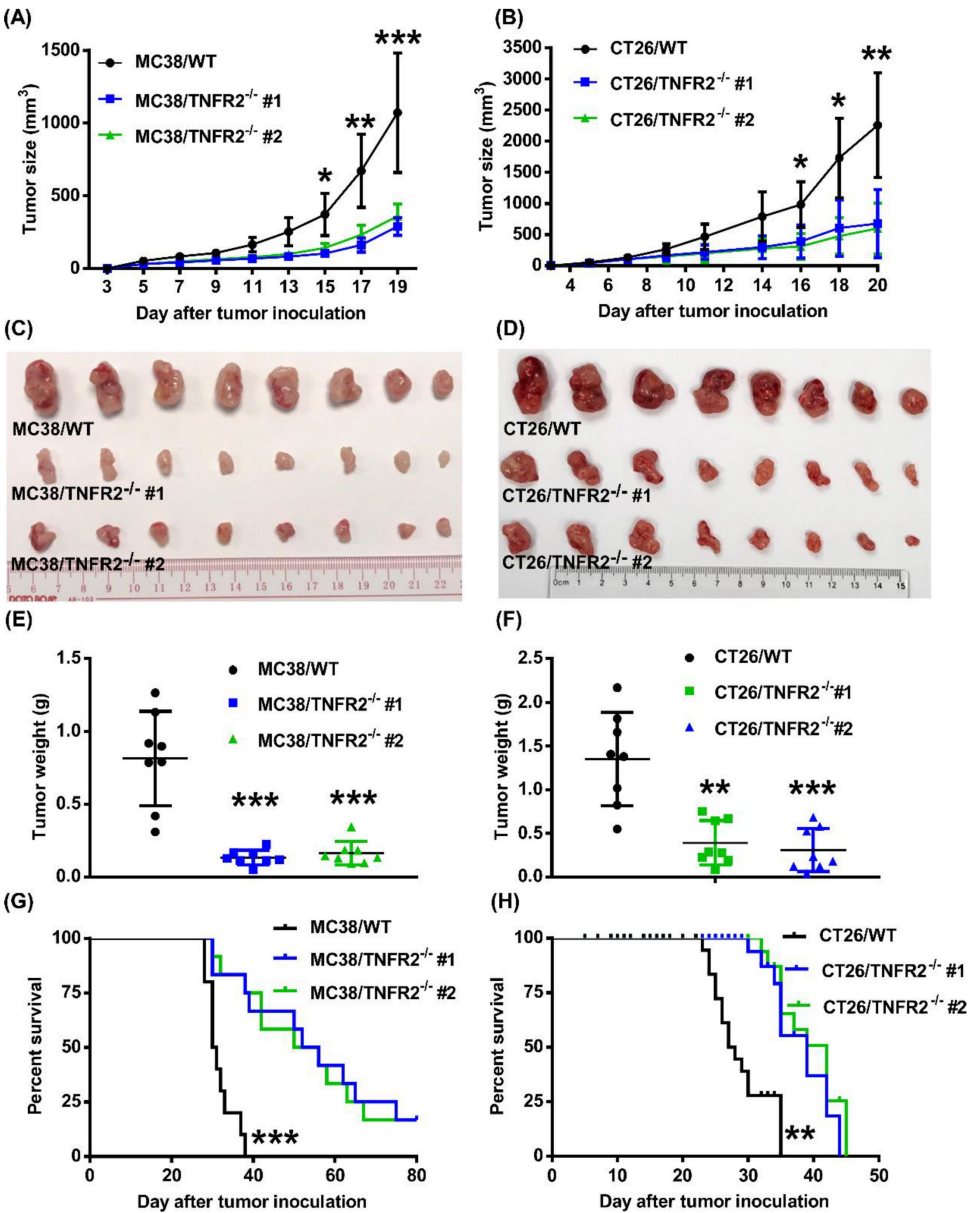
** Tumor growth is impaired in mice bearing TNFR2-deficient tumors.** MC38/WT cells or MC38/TNFR2^-/-^ cells (500,000 cells in 0.1 ml of PBS), and CT26/WT cells and CT26 TNFR2^-/-^ cells (200,000 cells in 0.1 ml of PBS), were inoculated in the right flank of C57BL/6 mice or BALB/c mice, respectively. The growth of the tumor was monitored, and on day 19 (MC38) or 20 (CT26) of tumor inoculation, mice were sacrificed, and the weight of the tumors was determined. In another set of experiments, the survival of tumor-bearing mice was examined. **(A and B)**: the tumor growth curve of cancers. **(C and D)**: Images and **(E and F)** weight of tumors. Data (means ± SEM, *N* = 8) shown in **(A, B, E and F)** are representatives of three separate experiments with similar results. **(G and H)** Survival curve of tumor-bearing mice. Data are pooled from two independent experiments (*N*=11-15 mice). By comparison with indicated groups, *P < 0.05, **P < 0.01, ***P < 0.001.

**Figure 5:**
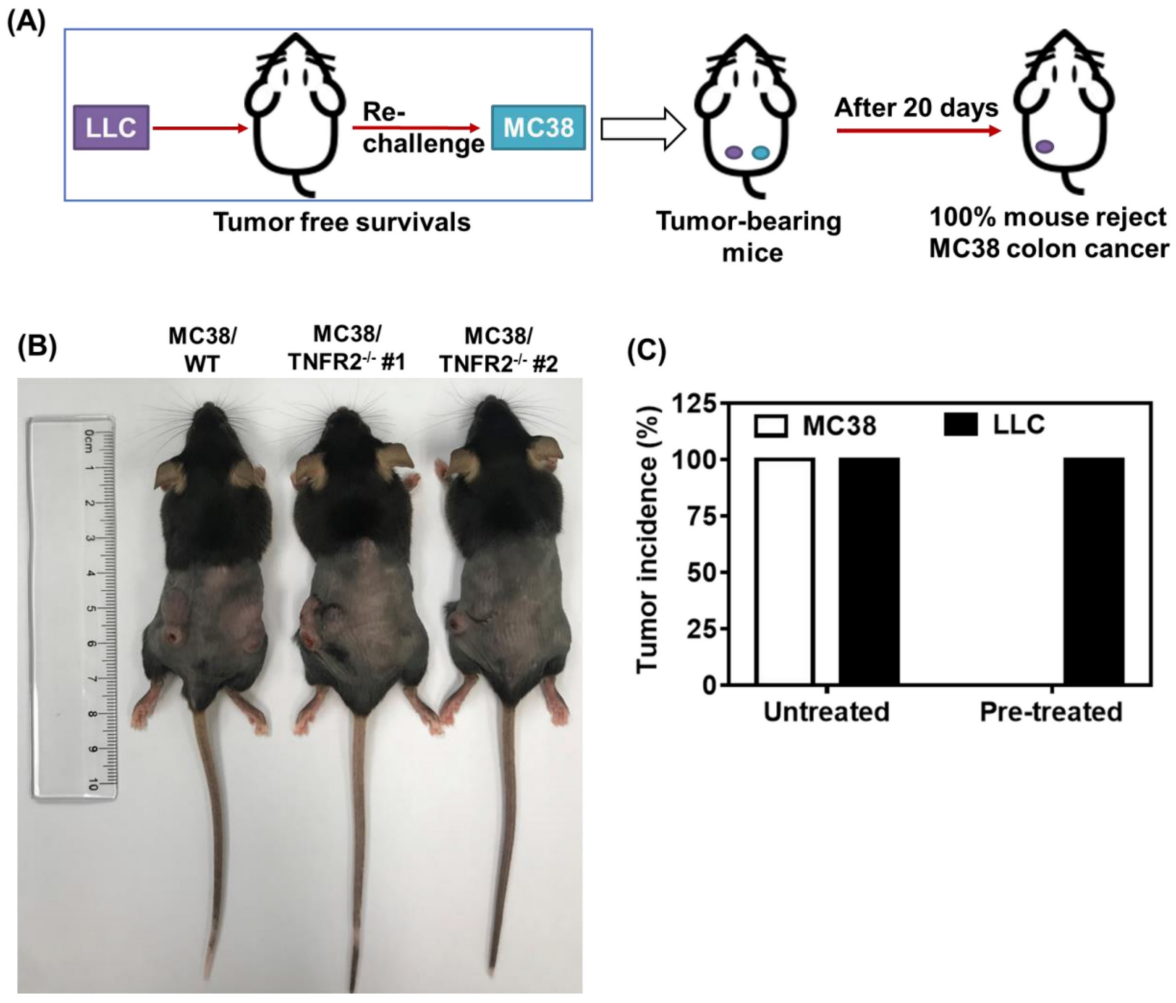
** Mice bearing TNFR2-deficient MC38 tumors develop tumor-specific immunity.** WT C57BL/6 mice were inoculated with TNFR2-deficient MC38 tumor cells as described in Figure 4 legend. After 100 days after initial tumor inoculation, the tumor-free surviving mice were reinoculated with MC38/WT tumor cells into the right flank and LLC tumor cells into the left flank, as schematically shown in **(A)**. As a control, normal mice of the same age were also inoculated with MC38/WT tumor cells to the right flank and LLC tumor cells into the left flank in the same manner. **(B)** The image of normal mice and surviving mice on day 20 after re-challenging tumor cells. **(C)** The percentage of tumor incidence on normal mice and surviving mice on day 20 after re-challenge of tumors (*N* = 5~7).

To examine the *in vivo* effects of TNFR2 ablation on the development of MC38 and CT26 colon cancers, we subcutaneously inoculated MC38/TNFR2^-/-^ and control cells (MC38/WT, 500,000 cells) into the right flank of C57BL/6 mice, or CT26/TNFR2^-/-^ and control cells (CT26/WT, 200,000 cells) into the right flank of BALB/c mice, respectively. The result showed that, although there was no effect on the initial tumor formation, deficiency of TNFR2 resulted in marked impairment of tumor growth (Figure 4A-F). On day 19 or 20 of inoculation, tumor sizes of TNFR2-deficient MC38 cell lines or CT26 cell lines were marked smaller (Figure 4A-B, *P* < 0.01-0.001), and the tumor image was shown in Figure 4C-D. Accordingly, the weights of TNFR2-deficient MC38 tumors and CT26 tumors were reduced by 83-85% and 71-77% (Figure 4E-F, *P* < 0.01-0.001), respectively. Furthermore, mice bearing TNFR2-deficient tumor cells survived longer significantly (Figure 4G-H, *P* < 0.01-0.001). More importantly, about 15% of MC38/TNFR2^-/-^ tumors were completely regressed within 28 days after inoculation. These tumor-free mice were alive for 80 days, whereas mice bearing W.T. tumor succumbed to tumor burden within 40 days after tumor inoculation (Figure 4G). To investigate whether the tumor-free mice developed long-term MC38 tumor-specific immunity, at 80 days after the tumor inoculation, the surviving mice were reinoculated subcutaneously with MC38/WT tumor cells into the right flanks, and WT LLC tumor cells were inoculated into their left flanks. As controls, both WT LLC tumor cells and MC38/WT tumor cells were inoculated into untreated mice in the same manner (as schematically illustrated in Figure 5A). As expected, all mice (100%) in the control group developed measurable MC38 and LLC tumors by day five after inoculation, including LLC tumors in those surviving mice. However, none of surviving mice developed MC38 colon tumors (Figure 5B-C). These results indicate that deficient in TNFR2 on tumor cells induced the development of long-term tumor antigen-specific immunity.

### Serum levels of sTNFR2 in mice inoculated with TNFR2-deficient tumor cells are reduced

**Figure 6:**
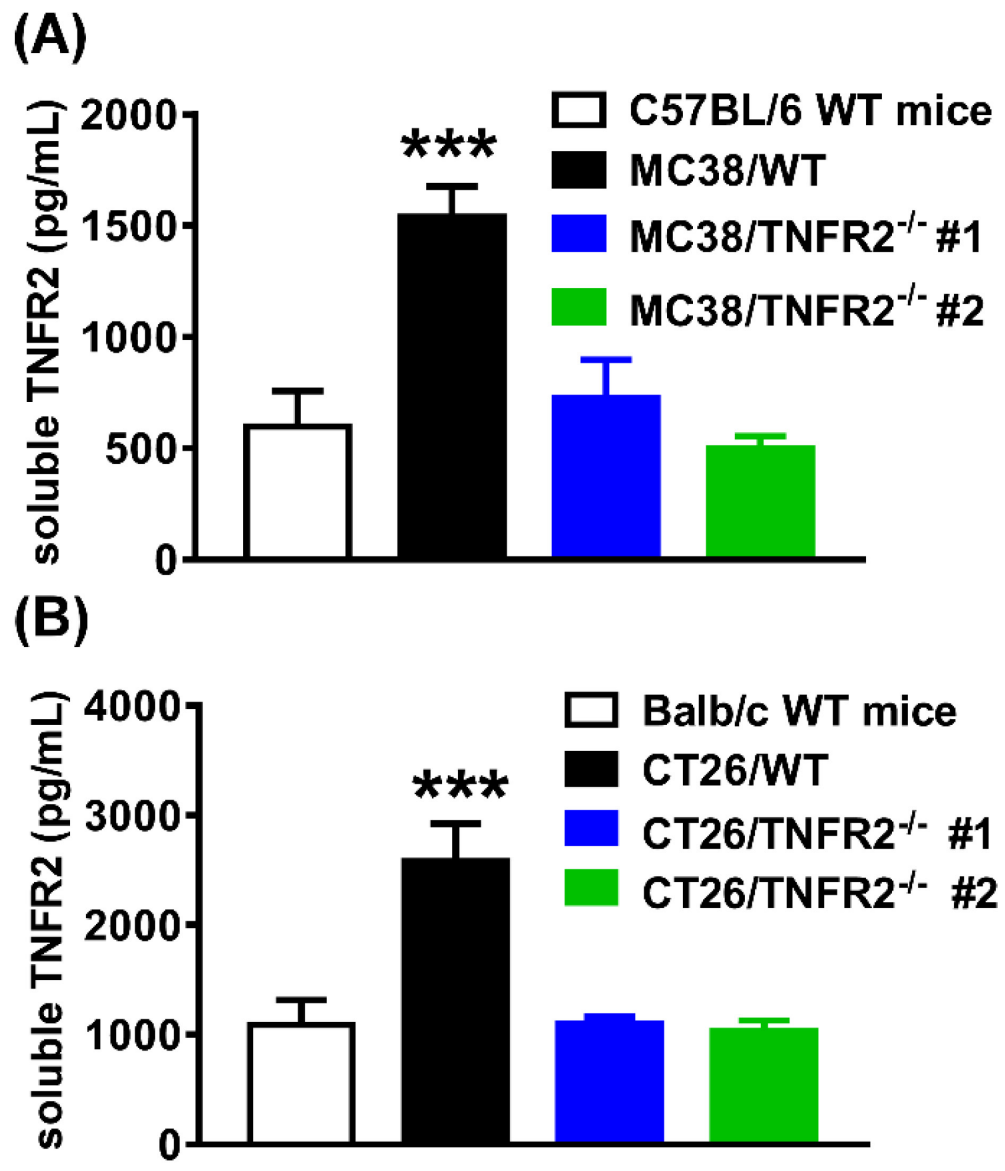
** Reduced the soluble TNFR2 in the serum of mice bearing TNFR2-deficient tumors.** Mice were inoculated with different tumors as described in Figure 4 legend. To generate the same size W.T. and TNFR2-deficient tumors, TNFR2-deficient cells were inoculated 12 days before inoculation of W.T. cells. On day 19 (MC38/WT) or 20 (CT26/WT) after the tumor inoculation, the serum was collected from the tumor-bearing mice, and the levels of soluble TNFR2 were measured by ELISA. The serum levels of soluble TNFR2 in MC38/WT and MC38/TNFR2^-/-^ tumor-bearing mice were shown in **(A)** and in CT26/WT and CT26/TNFR2^-/-^ tumor-bearing mice shown in **(B)**. Data (mean ± SEM, *N* = 8) shown are representative of three separate experiments with similar results. ***P < 0.001.

The elevation of soluble TNFR2 (sTNFR2) is correlated with the poor prognosis of tumor patients^1^,^25^-^27^. Since sTNFR2 levels were increased in the culture supernatant of MC38 and CT26 cells (Supplementary Figure S3) and the plasma of MC38 and CT26 tumor-bearing mice (Supplementary Figure S4) in a time-dependent manner, we thus hypothesized that tumors might be a source of sTNFR2. To test this, we generated the same size of W.T. and TNFR2-deficient tumors (about 1000 mm^3^ for MC38 tumor and 2000 mm^3^ for CT26 tumor) by inoculating TNFR2-deficient cells 12 days before the injection of W.T. cells. On day 19 (MC38/WT) or 20 (CT26/WT) days after tumor inoculation, the levels of sTNFR2 in serum were determined. The results showed that the levels of sTNFR2 were markedly enhanced by greater than 2-3 folds in mice bearing W.T. tumors, compared to tumor-free normal mice (Figure 6A-B, *P* < 0.001). Interestingly, sTNFR2 levels in mice bearing TNFR2-deficient tumors were markedly lower than that in mice bearing W.T. tumors (*P* < 0.001) and were close to that in tumor-free mice (Figure 6A-B). These data suggest that tumors may be the major, if not solely, source of upregulating part of sTNFR2 in tumor-bearing individuals.

### Increased infiltration of IFNγ^+^CD8^+^ CTLs in tumor deficient in TNFR2

**Figure 7:**
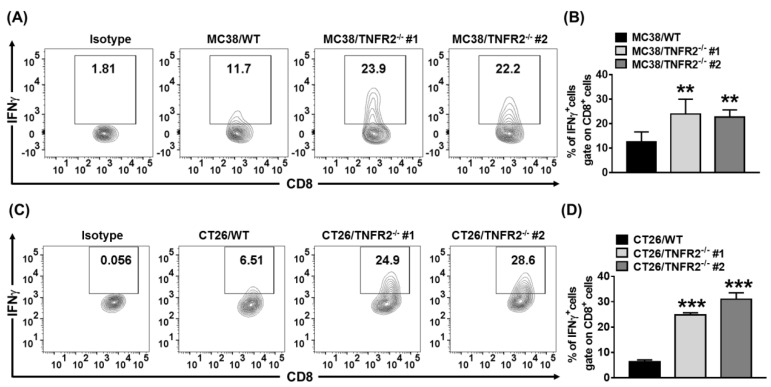
** Increased tumor infiltrating INFγ^+^CD8^+^ CTLs in mice bearing TNFR2-deficient tumors.** Same size W.T. or TNFR2-deficient MC38 or CT26 tumors were generated as described in Figure 6 legend. On day 19 (MC38) or 20 (CT26) after W.T. tumor inoculation, all mice were sacrificed. The proportion of INFγ^+^ cells in live CD45^+^TCRβ^+^ CD8^+^ cells in tumor tissue was analyzed by FACS**. (A and C)** show typical FACS plots. **(B and D)** show the summary of the proportion of INFγ^+^ cells in tumor-infiltrating CD8^+^ cells. Data (mean ± SEM, *N* = 6) shown are representatives of three separate experiments with similar results. As compared with WT group, *P < 0.05, **P < 0.01, ***P < 0.001.

sTNFR2 is a potent immunosuppressive mediator^28^. Our results also showed that etanercept, a soluble form of TNFR2^29^, could inhibit CD4^+^ T cell proliferation (Figure S5). Thus, the reduced levels of sTNFR2 may result in the mobilization of antitumor immune responses. To test this, we examined Th1 cells present in the same size of W.T. and TNFR2^-/-^ tumors (as described in 3.6). On day 19 (MC38/WT) or day 20 (CT26/WT) after tumor inoculation, the tumor-infiltrating leukocytes were analyzed. The result showed that the proportion of live CD45^+^, CD45^+^TCRβ^+^, CD45^+^TCRβ^+^CD4^+^, and CD45^+^TCRβ^+^CD8^+^ cells were increased in TNFR2^-/-^ tumor tissues (Figure S7). More importantly, the proportion of tumor-infiltrating IFNγ-expressing CD8^+^ CTLs was increased by 2-4 folds in TNFR2-deficient tumors, compared with those in W.T. tumors (Figure 7, *P* < 0.01-0.001). Thus, our data indicate that deficiency of TNFR2 on MC38 and CT26 tumor cells induced potent CD8^+^ T cells mediated antitumor immune responses.

## Discussion

It has been reported that TNF is expressed in both human and mouse colon cancer cells (such as HCT116 and CT26)^30^, and TNF has the capacity to promote the proliferation of colon cancer cells^31^. Further, it was shown that the increased expression of TNF is associated with advanced colorectal cancer stages^30^,^32^. In addition, in the presence of effector cells (macrophages) or active complements, anti-TNF treatment with infliximab markedly suppressed the survival of colon cancer cells^31^. Such an effect of TNF on tumor cells is likely mediated by TNFR2. In fact, the functional consequences of TNFR2 expression on mouse tumor cells were reported previously. For example, it was shown that knockdown of TNFR2 by shRNA rendered mouse Lewis Lung Carcinoma (LLC) more susceptible to TNF-induced apoptosis, accompanied by the down-regulation of Vegfa, Hgf, Plafg, and Cxcr4 expression, while the growth of such LLC cells in normal mice was normal^33^. In this study, the efficiency of TNFR2 knockdown was ~90%, while the remaining TNFR2 may still be able to support the *in vivo* growth of tumor cells. Furthermore, the surface expression levels of TNFR2 on LLC cells maintained in our own lab were much lower (data not shown) than that expressed by CT26 and MC38 tumor cells. That was why we decided to examine the functional implications of TNFR2 by genetically ablating TNFR2 on CT26 and MC38 tumor lines with CRISPR/Cas9 technology. The results of our study clearly showed that the deficiency of TNFR2 potently impaired the *in vitro* and *in vivo* growth of the tumor.

Previously, it was shown that overexpression of TNFR2 in tumor cells promotes cell proliferation and clone formation^9^. We thus also created TNFR2-overexpressing MC38 and CT26 colon cancer cells by infecting Plvx-IRES-ZsGreen1-TNFR2 lentivirus vector. Plvx-IRES-ZsGreen1 and TNFR2 cDNA were digested by EcoRI and BamHI and then were ligated by Quick T4 DNA ligase to construct the Plvx-IRES-ZsGreen1-TNFR2 overexpression plasmid. Unexpectedly, overexpression of TNFR2 in MC38 and CT26 did not further promote the proliferation of tumor cells. One possible reason is that the original levels of TNFR2 have already rendered the maximal effect on the survival and proliferation of these two lines of cancer cells.

Once cleaved, membrane-bound TNFR2 becomes soluble TNFR2 (sTNFR2). It is well known that sTNFR2 is immunosuppressive and this notion could be verified by our experimental result showing that etanercept, a soluble form of TNFR2^29^, had the capacity to inhibit the proliferation of CD4^+^ T cells (Figure S5). Therefore, the decreased levels of sTNFR2 in mice bearing TNFR2-deficient tumor was most likely attributable to the activation and expansion of tumor infiltrating CD8 CTLs.

Unlike TNFR2, TNFR1 is expressed by almost all types of cells except erythrocytes. With a death domain, TNFR1 signaling induces the cascade related to apoptosis^34^. In this study, we also determined the TNFR1 surface expression (using an anti-TNFR1 antibody, Serotec, HM104) by FACS and the soluble TNFR1 levels in the culture supernatant using ELISA kit. Our results showed that the TNFR1 was almost undetectable on the surface of MC38 and CT26 tumor cells. However, the mRNA level of TNFR1 was detectable by real-time PCR by these cancer cells. Furthermore, TNFR2 deletion did not affect the mRNA expression of TNFR1 (data shown in Figure S8). This result was also similar to that reported by others in human colorectal cancer cell^35^, which indicated that the silencing TNFR2 expression did not affect TNFR1 mRNA level. Further, the soluble TNFR1 levels were much lower than sTNFR2 (data not shown). Nevertheless, elevated levels of sTNFR1 were found in the supernatant of tumor cell culture medium and in the serum of tumor-bearing mice (data not shown), which is consistent with clinical reports^36^. Thus, sTNFR1 may also be attributable to tumor immunosuppression, and this possibility should be addressed in future studies.

Aberrant activation of the PI3K/AKT signaling pathway facilitates a variety of cellular activities such as proliferation, cell cycle progression, aggressiveness, and chemoresistance in many cancers, especially in CRC^37^. Previous studies also revealed that TNFR2 neutralizing antibody could block the activation of PI3K/AKT and ERK signal pathway in cholangiocarcinoma cells^5^, and TNFR2 promoted the growth of human SW480, COLO205, and HT29 colorectal cancer cells through PI3K/AKT signaling pathway or activation of STAT3^9^,^35^. The immunohistochemistry (IHC) study showed that TNFR2 was positively associated with Ki67 expression in CRC tissues. Further, the western blot analysis found that TNFR2 promoted Ki67 expression in CRC cells via the PI3K/AKT signaling pathway^9^. Thus, the clinical evidence also supports the notion that TNFR2 promotes the growth of CRC. In this study, we found that deficiency of TNFR2 reduced the phosphorylation of AKT (Figure 3A). In contrast, the phosphorylation of ERK, JNK, STAT3, and NF-κB was not inhibited by TNFR2 deficiency (data not shown), which was likely caused by intact TNFR1 signal, since knockout of TNFR2 did not affect the expression of TNFR1 (data shown in Figure S8). Our data indicate that the inhibition of PI3K-AKT signaling pathway may be a mechanism underlying the growth inhibition found in TNFR2-deficient tumor cells.

Autophagy occurs after nutrient deprivation or following chemotherapy in cancer cells^38^,^39^. The impact of autophagy on cancer progression is controversial, and whether the activation of autophagy in tumor cells actually causes death or represents a survival mechanism remains unclear. Autophagy also serves to remove damaged and potentially harmful organelles, thereby supporting cell survival. On the other hand, there is conclusive evidence that prolonged over-activation of the autophagosomal/lysosomal pathway can lead to autophagic cell death^40^. A previous study showed that knockdown of RAB25 promotes autophagy and inhibits cell growth in ovarian cancer cells^41^. Further, autophagy induced by inhibiting the PI3K/Akt/mTOR pathway causes growth inhibition and death of cancer cells^42^. In the present study, deficiency of TNFR2 promoted autophagy, suggesting that the tumor cells underwent stress due to the deficiency of TNFR2 signaling.

Taken together, our study clearly shows that deficiency of TNFR2 on mouse MC38 and CT26 colon cancer cells could markedly impair the growth of the tumor, which may be attributable to the inhibition of AKT signaling pathway, and activation of autophagy and mobilization of antitumor Th1 responses due to reduction of sTNFR2. Our data suggest that the inhibition of tumor growth likely resulted from the direct effect on tumor cells as well as from the mobilization of anti-tumor immune responses. Our data provide further experimental evidence to support the notion that blockage of TNFR2 may represent a novel strategy in cancer immunotherapy.

## Supplementary Material

Supplementary results and figures.
