# Supplementary material for: TNFR2 deficiency impairs the growth of mouse colon cancer
Source: Int J Biol Sci. 2023 Jan 22;19(4):1024–35. doi: 10.7150/ijbs.72606 (PMC10008691; doi:10.7150/ijbs.72606)
Supplement: Supplementary file 1 — Supplementary results and figures. [file ijbsv19p1024s1.pdf]

TNFR2 deficiency impairs the growth of mouse colon cancer

Ping Li[1], Yang Yang[1], Xinyu Yang[1], Yifei Wang[1], Chon-Kit Chou[1], Mengmeng Jiang[1], Jingbin Zheng[1], Fengyang Chen[1], Xin Chen[1,2,3,*]

[1] State Key Laboratory of Quality Research in Chinese Medicine, Institute of Chinese Medical Science, University of Macau, Macau, 999078, P.R. China.

[2] Department of Pharmaceutical Sciences, Faculty of Health Sciences, University of Macau, Macau, China.

[3] MoE Frontiers Science Center for Precision Oncology, University of Macau, Macau, China.

Contact information:

* Corresponding author

Xin Chen E-mail: xchen@um.edu.mo

Results

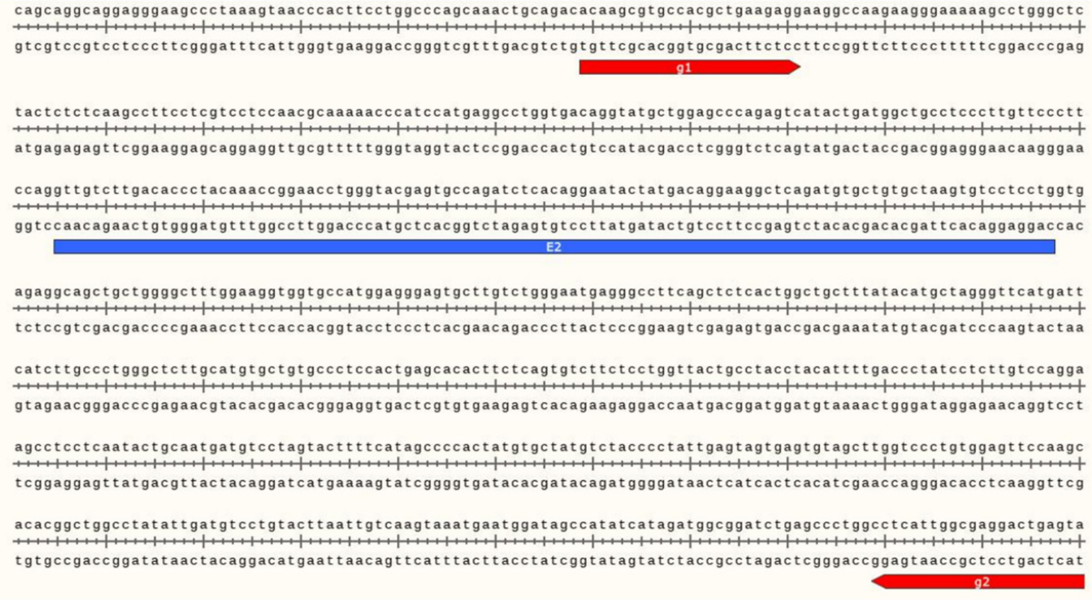

Figure S1. Two TNFR2-targeted sgRNAs.

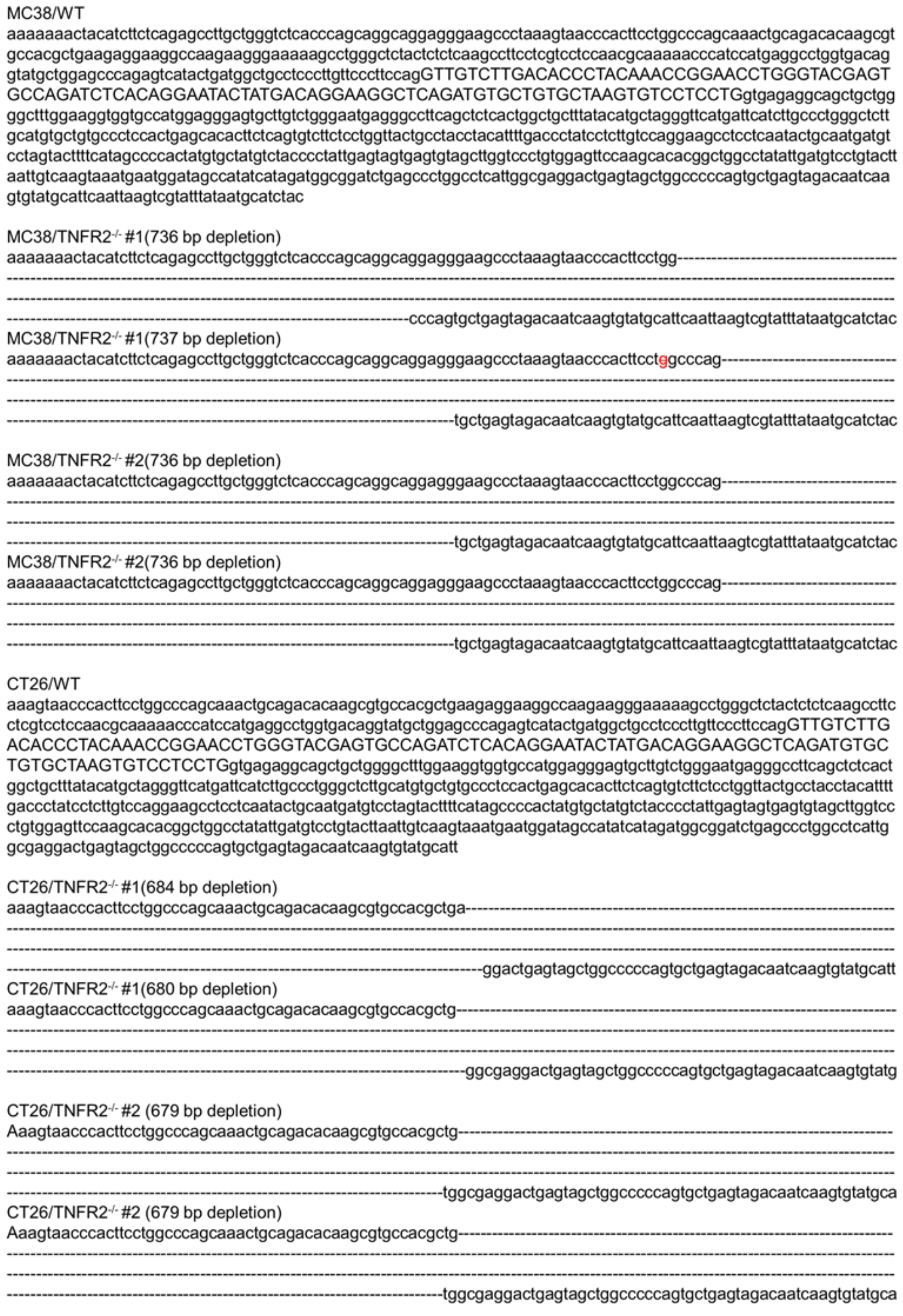

Figure S2. Generation of TNFR2-knockout MC38 and CT26 cell lines using the CRISPR/Cas9 system. Two TNFR2$^{-/-}$ cell lines were established from MC38 and CT26 cells, respectively. The deleted sequences in the TNFR2$^{-/-}$ #1 and TNFR2$^{-/-}$ #2 cell lines are presented.

(A)

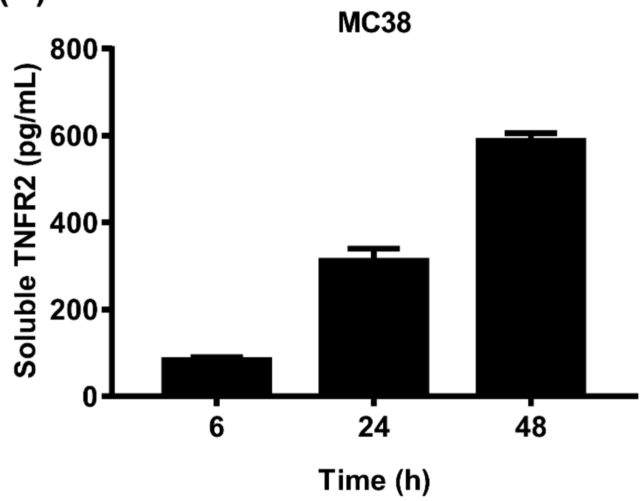

(B)

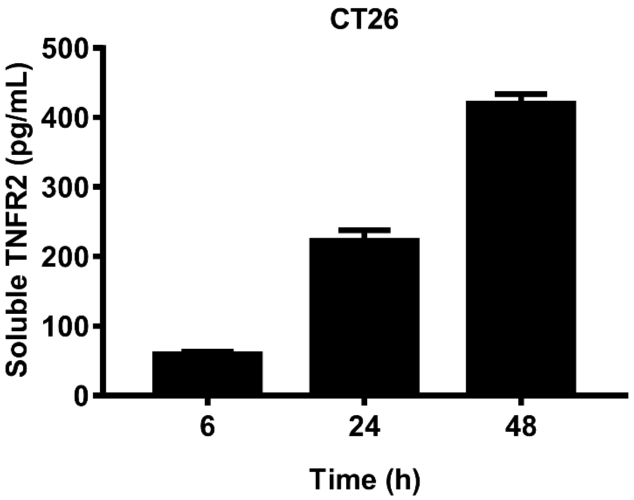

Figure S3. Increasing the soluble TNFR2 level in the culture supernatant with a time-dependent manner. MC38 or CT26 tumor cells were cultured with 24-well plate with a concentration of 200,000 cells/mL, each well with 0.5 mL cell suspension. After 6, 24, and 48 h, the soluble TNFR2 of MC38 (A) and CT26 (B) in the supernatant was determined by the ELISA kit according the manufacturer's instructions, respectively. Data (mean ± SEM, $N = 4$) shown are representatives of at least three separate experiments with similar results.

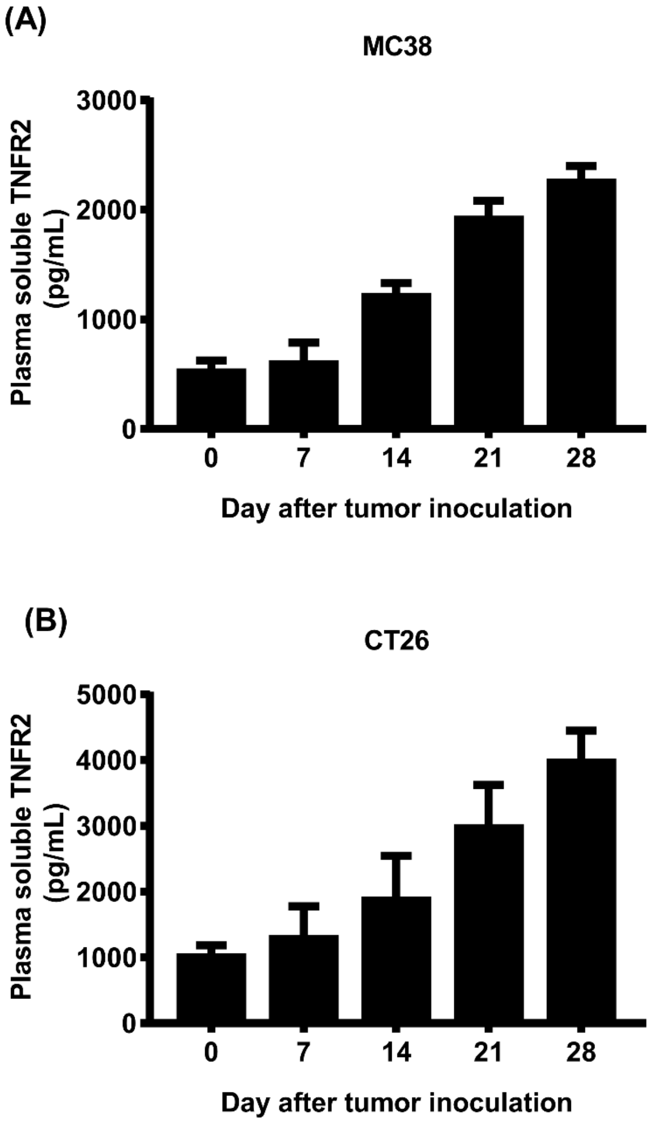

Figure S4. Increasing the soluble TNFR2 level in the plasma of MC38 and CT26 tumor-bearing mice with a time-dependent manner. C57BL/6 mice were inoculated in the right flank with MC38/WT cells (500,000 cells in 0.1 ml of PBS) and Balb/c mice were inoculated in the right flank with CT26/WT (200,000 cells in 0.1 ml of PBS), respectively. As the time indicated after the tumor inoculation, the plasma were collected from the tumor-bearing mice, and the soluble TNFR2 in the plasma of different tumor-bearing mice as indicated were measured by ELISA. (A) The soluble TNFR2 from plasma of MC38 tumor-bearing mice. (B) The soluble TNFR2 from plasma of MC38 tumor-bearing mice. Data (mean ± SEM, *N* = 4) shown are representative of three separate experiments with similar results.

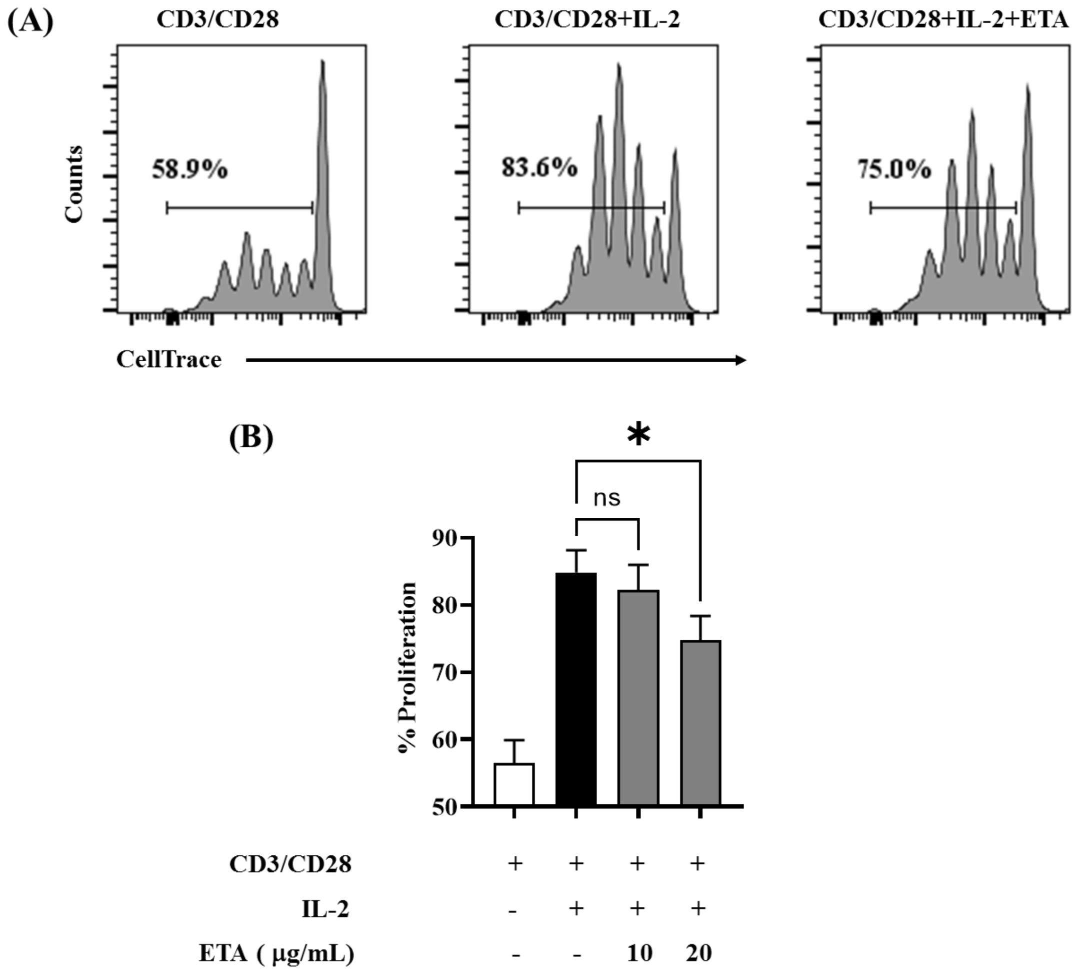

Figure S5. The effects of etanercept (ETA) on the expansion of CD4⁺ T cells induced by CD3/CD28/IL-2. Purified $CD4^+$ cells were labeled with CellTrace™ Violet and cultured in medium containing CD3/CD28 in the presence or absence of IL-2. The cells were treated with the indicated concentrations of ETA. After 3 days, the percentage of replicating $CD4^+$ T cells, based on the dilution of the CellTrace™ Violet signal, was analyzed by fluorescence-activated cell sorting (FACS), by gating of $CD4^+$ cells. (A) Typical FACS plots and (B) ETA inhibited $CD4^+$ T cells proliferation induced by CD3/C28/IL-2. Data (means ± SD, N = 3) shown are representatives of 2 separate experiments with similar results. *$P < 0.05$.

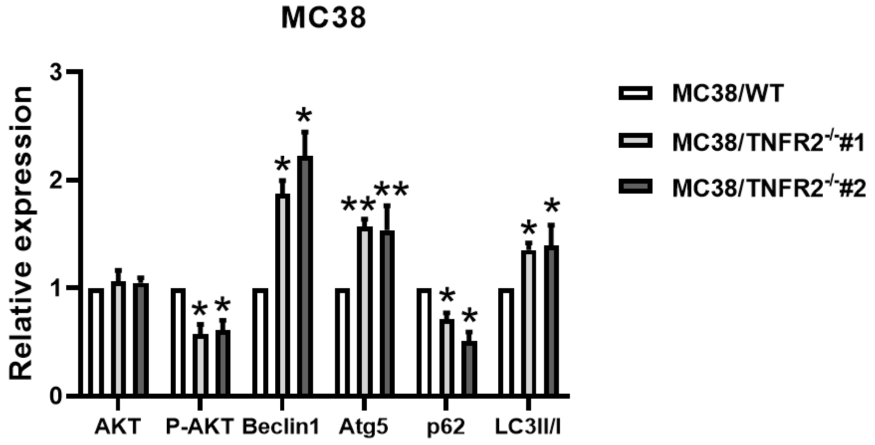

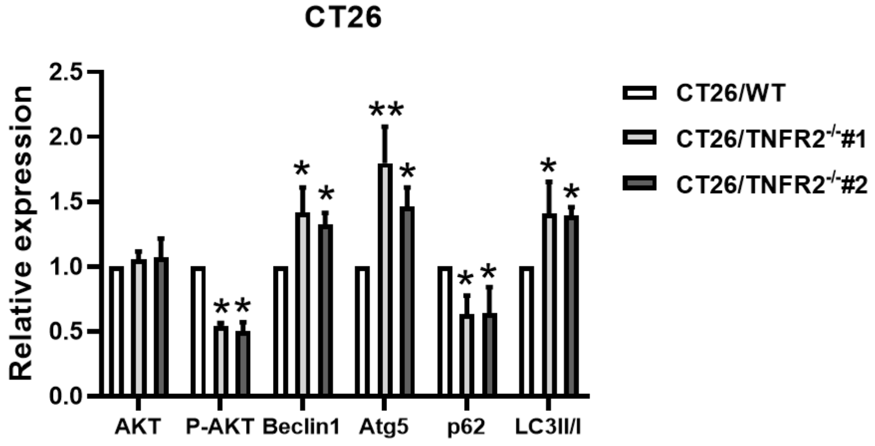

Figure S6. The deficiency of TNFR2 reduces phosphorylation of AKT and promotes autophagy. Western blot analysis of P-AKT, AKT, and β-actin; LC3, Beclin 1, p62, Atg5, and GAPDH. The lysates derived from MC38/WT, MC38/TNFR2$^{-/-}$#1, MC38/TNFR2$^{-/-}$#1; CT26/WT, CT26/TNFR2$^{-/-}$#1, and CT26/TNFR2$^{-/-}$#2 cells were immunoblotted with a panel of antibodies specific for P-AKT, AKT, and β-actin; LC3, Beclin 1, p62, Atg5, and GAPDH (a loading control), respectively. Data (mean ± SEM, N = 3) shown are representative of three separate experiments with similar results. As compared with W.T. group, *P < 0.05, **P < 0.01.

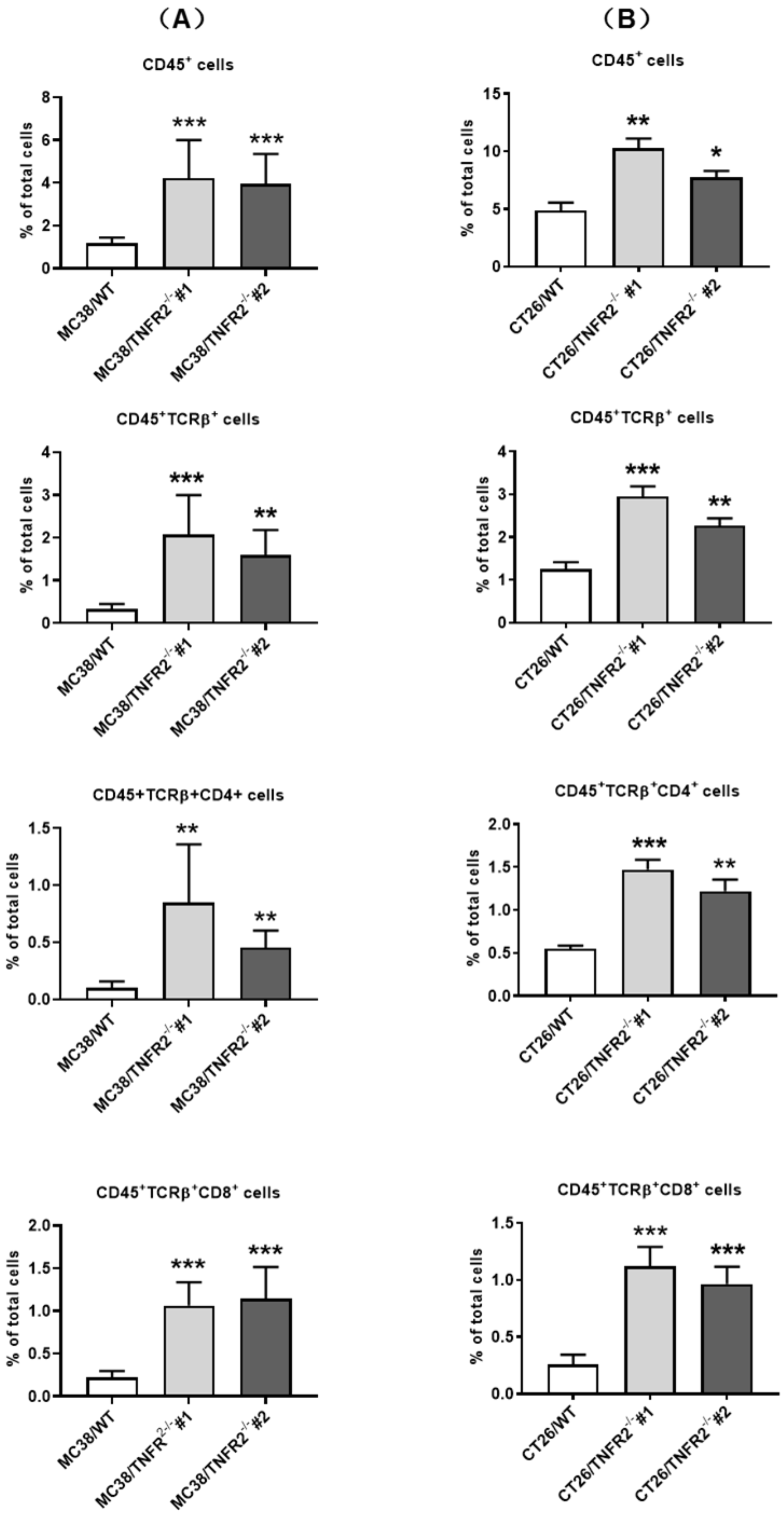

Figure S7. Increased the proportion of live CD45^{+}, CD45^{+}TCRβ^{+}, CD45^{+}TCRβ^{+}CD4^{+}, CD45^{+}TCRβ^{+}CD8^{+} cells were increased in TNFR2$^{-/-}$ tumor

tissues. Same size WT or TNFR2-deficient MC38 or CT26 tumors were generated as described in Figure 6 legend. On day 19 (MC38) or 20 (CT26) after WT tumor inoculation, all mice were sacrificed. The proportion of live $CD45^+$, $CD45^+TCR\beta^+$, $CD45^+TCR\beta^+CD4^+$, $CD45^+TCR\beta^+CD8^+$ in tumor tissue analyzed by FACS. (A) MC38 tumor model, (B) CT26 tumor model. Data (mean ± SEM, $N = 6$) shown are representative of three separate experiments with similar results. As compared with W.T. group, *$P < 0.05$, **$P < 0.01$, ***$P < 0.001$.

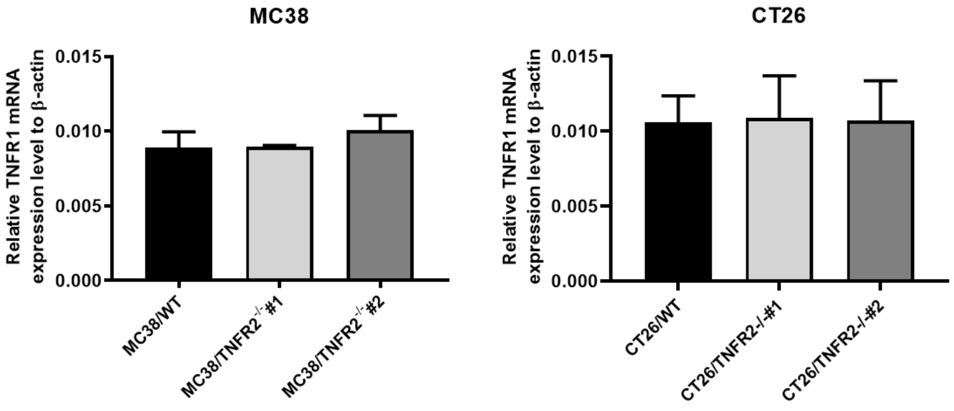

Figure S8. Real-time PCR analysis of TNFR1 derived from W.T. and $TNFR2^{-/-}$ cell lines.
